# The Multi-Faceted Effect of Curcumin in Glioblastoma from Rescuing Cell Clearance to Autophagy-Independent Effects

**DOI:** 10.3390/molecules25204839

**Published:** 2020-10-20

**Authors:** Larisa Ryskalin, Francesca Biagioni, Carla L. Busceti, Gloria Lazzeri, Alessandro Frati, Francesco Fornai

**Affiliations:** 1 Department of Translational Research and New Technologies in Medicine and Surgery, University of Pisa, Via Roma 55, 56126 Pisa, Italy; larisa.ryskalin@unipi.it (L.R.); gloria.lazzeri@unipi.it (G.L.); 2 I.R.C.C.S. Neuromed, Via Atinense 18, 86077 Pozzilli, Italy; francesca.biagioni@neuromed.it (F.B.); carla.busceti@neuromed.it (C.L.B.); alessandro.frati@uniroma1.it (A.F.)

**Keywords:** curcuma longa, natural polyphenols, neuroprotection, anti-cancer effects, glioblastoma stem-like cells, autophagy

## Abstract

The present review focuses on the multi-faceted effects of curcumin on the neurobiology glioblastoma multiforme (GBM), with a special emphasis on autophagy (ATG)-dependent molecular pathways activated by such a natural polyphenol. This is consistent with the effects of curcumin in a variety of experimental models of neurodegeneration, where the molecular events partially overlap with GBM. In fact, curcumin broadly affects various signaling pathways, which are similarly affected in cell degeneration and cell differentiation. The antitumoral effects of curcumin include growth inhibition, cell cycle arrest, anti-migration and anti-invasion, as well as chemo- and radio-sensitizing activity. Remarkably, most of these effects rely on mammalian target of rapamycin (mTOR)-dependent ATG induction. In addition, curcumin targets undifferentiated and highly tumorigenic GBM cancer stem cells (GSCs). When rescuing ATG with curcumin, the tumorigenic feature of GSCs is suppressed, thus counteracting GBM establishment and growth. It is noteworthy that targeting GSCs may also help overcome therapeutic resistance and reduce tumor relapse, which may lead to a significant improvement of GBM prognosis. The present review focuses on the multi-faceted effects of curcumin on GBM neurobiology, which represents an extension to its neuroprotective efficacy.

## 1. Introduction

Curcumin is a natural polyphenol extracted from the rhizome of *Curcuma longa*; it is also known as turmeric [1] and was introduced to Europe in the 14th century as a culinary spice [2]. This natural compound has been used for centuries in the traditional Indian Ayurvedic and Chinese medicine for treating respiratory and liver disorders, infections, allergies, and rheumatisms [3]. Nowadays, curcumin is widely used due to its multiple biological activities, encompassing anti-inflammatory, anti-oxidant, scavenging reactive oxidative species (ROS), regulation of mitochondrial homeostasis, stem cell modulation, and neurogenesis [4]. Hence, curcumin may be useful in a wide range of human diseases, encompassing cardiovascular [5,6], inflammatory [7,8], and metabolic disorders [9,10,11].

In particular, in the last decades, phytochemicals, and especially curcumin, have gathered increasing interest in both experimental and pre-clinical studies for their beneficial effects on the central nervous system (CNS). Among dietary polyphenolic compounds, curcumin appears as a useful agent for adjunct therapy in a variety of neurogenerative disorders (NDDs) [4,12]. In fact, curcumin appears to exert multiple neuroprotective effects through its strong anti-oxidant, anti-inflammatory, and anti-protein aggregation properties [13]. Moreover, increasing evidence argues for curcumin’s anti-proliferative, anti-invasive, anti-angiogenic, and chemo-preventive potential, which suggest a potential usefulness in neoplasms [14]. In line with this, numerous studies demonstrated the chemo- and radio-sensitizing effects of curcumin in various cancers, including glioblastoma multiforme (GBM) [15,16,17,18,19]. 

The pleiotropic therapeutic activities of curcumin rely on its ability to influence various signaling pathways [20]. In fact, curcumin operates in multiple ways by modulating the activity of kinases and transcription factors as well as by regulating the expression of genes involved in cell survival and malignant transformation [20]. The ability of curcumin to target different pathways is bound to its unique and complex chemical structure. In fact, this natural polyphenol, also known as diferuloylmethane (1,7-bis (4-hydroxy-3-methoxyphenyl)-1,6-hepadiene-3,5-dione; C_21_H_20_O6), possesses three reactive functional groups, namely 1,3-diketone moiety and two phenolic groups. The latter are key for curcumin’s multiple biological activities [21], which render this natural bioactive compound an attractive and flexible therapeutic option that was tested in experimental models of neurodegeneration and glioblastoma multiforme (GBM). 

It is remarkable that the beneficial effects induced by curcumin in GBM are tightly associated with neuroprotective activities in a variety of neurological insults. Within this frame, the onset and progression of GBM is considered for many instances as reminiscent of an accelerated variant of neurodegeneration. This is key to comprehend the common background based on mammalian target of rapamycin (mTOR), which is a ubiquitously expressed serine-threonine kinase, which controls major processes such as cell growth, proliferation, metabolism, and protein degradation pathways such as autophagy (ATG). Remarkably, mTOR dysfunction and related ATG alterations are described in a variety of neurodegenerative conditions while being powerfully manifest in GBM [22,23].

A brief overview of the wide range of neuroprotective effects of curcumin in neurodegeneration is provided here to link such an activity to curcumin-induced anti-cancer effects in GBM. Such an approach allows discussing the multi-faceted effects of curcumin on GBM neurobiology. Thus, we provide evidence on how curcumin inhibits GBM growth and infiltration through the modulation of ATG-dependent pathways. In this respect, we specifically focus on how curcumin-activated ATG in turn modulates stem-like properties of GBM cancer stem cell (GSCs), while forcing them to undergo differentiation, thus limiting GBM infiltration and volume growth. Novel insights into the mechanisms by which curcumin impacts GSCs tumorigenicity, and thus a GBM malignant phenotype, will contribute to developing novel therapeutic strategies while improving GBM poor prognosis. The last part of the review is focused on the potential of curcumin as a disease-modifier of GBM neurobiology beyond its effect as an ATG inducer.

## 2. Pleiotropic Effects of Curcumin in the CNS: from Neuroprotection to Anti-Cancer Activities

Among a number of phytochemicals, curcumin has been widely investigated in experimental and clinical studies for its potential benefits to counteract oxidative stress, mitochondrial damage, synaptic dysfunction, neuro-inflammation, and the accumulation of aggregate-prone proteins, which are implicated in various NDDs, including Parkinson’s disease (PD) and Alzheimer’s disease (AD). Far from being independent, these effects converge into common metabolic pathways, which appear to be ubiquitously altered in most NDDs. This is independent of etiology and neuroanatomical site-specificity. This is the case of mTOR-dependent alterations of the ATG pathway, which appears as a recurring feature in NDDs [23,24,25].

Within this frame, it is remarkable that the beneficial effects of curcumin including anti-oxidant, anti-inflammatory, mitochondrial protection, and anti-amyloidogenic activities are bound to the modulation of mTOR-dependent ATG. In fact, by targeting the ATG pathway, curcumin breaks a vicious cycle, which is initiated by ATG defects and impaired proteostasis. This is worsened by protein misfolding, oxidative stress, and neuro-inflammation. All these events further boost the neurodegeneration cascade. A large body of evidence converges in demonstrating that curcumin supplementation confers neuroprotection by rescuing ATG. For instance, curcumin counteracts 6-OHDA-induced cell death in SH-SY5Y cells by enhancing the ATG-dependent clearance of damaged mitochondria and removal of excess ROS [26]. Curcumin also counteracts mitochondrial damage by enhancing mitochondria ATG (i.e., mitophagy), which selectively removes damaged and/or aged mitochondria [27]. As already demonstrated for physical exercise or caloric restriction, which can induce beneficial adaptations for metabolic homeostasis by ameliorating mitochondrial function [28], also, phytochemicals were shown to enhance mitochondrial function, which, in turn, is bound to the ATG-dependent orchestration of mitochondrial dynamics [29]. Furthermore, curcumin-dependent ATG activation suppresses the inflammatory response following neurological insults [30,31]. For instance, curcumin-treated transgenic AD mice show a marked decrease in pro-inflammatory cytokines (i.e., IL-1β, TNF-α, IL-6) levels and microglial activation, which are associated with brain injury and inflammatory processes [32,33]. In contrast, ATG inhibition abrogates the beneficial effects of curcumin [31]. Again, anti-fibrillogenic effects of curcumin in counteracting the aggregation of proteins such as tau, amyloid-beta, and α-synuclein, which occur in NDDs, rely on the very same mTOR inhibition, which in turn produces ATG activation [33,34,35]. For instance, treatment with curcumin reduces the pathological accumulation of mutated (A53T) α-synuclein in dopamine (DA)-containing SH-SY5Y cells through downregulation of the mTOR/p70S6K pathway and thus ATG recovery [36]. Similarly, curcumin counteracts the aggregation of prion protein [37], which besides classic prion disorders is a manifest of neurotoxicant-induced models of PD [38,39]. Since curcumin produces a strong activation of ATG, the potential beneficial effects of such a compound were tested in various ATG deficiencies. This is the case of GBM, which due to a marked mTOR up-regulation, features an occlusion of the ATG pathway. Thus, in further studies, the ability of curcumin in mediating neuroprotective effects through ATG induction took a center stage in cancer research, and particularly in GBM. 

According to the World Health Organization (WHO), GBM is the most aggressive and lethal primary brain tumor [40,41]. In fact, despite current advances in neurosurgery and radio- and chemotherapy, the prognosis is still dismal [42]. The biology of this neoplasm is complex. In particular, GBM carries numerous genetic and molecular alterations leading to aberrant signaling pathways that contribute to GBM pathogenesis [43,44]. Therefore, developing therapeutic approaches targeting multiple oncogenic signaling aberrations associated with GBM needs articulated compounds.

Curcumin is reported to produce anti-proliferative, anti-migration, and pro-apoptotic effects in experimental models of GBM [45,46,47,48] (Table 1).

**Figure 1:**
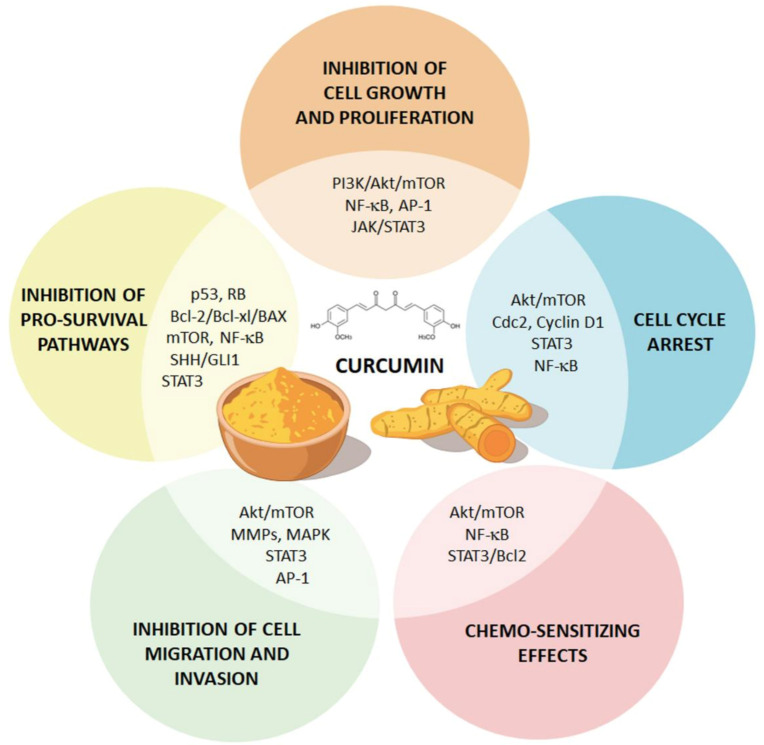
Curcumin modulates major glioblastoma multiforme (GBM)-associated signaling pathways. The cartoon summarizes the major effects of curcumin on GBM cells. In fact, curcumin was shown to broadly affect core-signaling pathways of GBM neurobiology. For instance, curcumin suppresses tumor growth by inhibiting tumor-promoting pathways (i.e., nuclear factor κB (NF-kB), phosphoinositide 3-kinases/Akt/mammalian target of rapamycin (PI3K/Akt/mTOR), Janus kinase/signal transducers and activators of transcription (JAK/STAT3) and mitogen-activated protein kinase (MAPK) pathways), while up-regulating major tumor-suppressing (i.e., p53 and p21, and caspase).

In fact, curcumin modulates the core-signaling pathways of GBM neurobiology, including the nuclear factor κB (NF-κB), activator protein-1 (AP-1), Janus kinase/signal transducers and activators of transcription (JAK/STAT), TP53, and RB, as well as mitogen-activated protein kinase/extracellular-signal-regulated kinase (MAPK/ERK) and phosphoinositide 3-kinases/Akt/mammalian target of rapamycin (PI3K/Akt/mTOR) and ATG pathways [44] (Figure 1).

Consistent with in vitro studies, beneficial effects of curcumin have been reported in several in vivo models of GBM. These encompass the inhibition of cell proliferation and matrix metalloproteinases (MMP)-dependent cell migration and invasion, thus resulting in decreased tumor volume along with increased survival time [49,59,60] (Table 2).

It is noteworthy that curcumin recently emerges as a promising adjunct therapy against GBM owing to its potential to target GSCs [50,51,52,61,62,63,64,65,66]. This represents a sub-population of cancer cells endowed with stem-like features, such as increased self-renewal, pluripotency, and clonogenic potential [22,39]. Notably, GSCs are responsible for GBM initiation, progression, tumor re-growth after surgical resection, and thus patient relapse [67]. GSCs are resistant to standard treatments [68], which contribute to an inauspicious prognosis for GBM patients. Therefore, strategies aimed at eradicating these cells hold great promises for developing novel therapeutic approaches against GBM.

Among various pathways, which sustain GSCs’ oncogenic properties and metabolism, mTOR-dependent ATG plays a seminal role. While finely tuned mTOR signaling is essential for normal CNS development, GSCs take advantage of an improper mTOR activity to fuel tumor growth and infiltration [69]. In fact, these cells feature a robust up-regulation of mTOR, and thus a marked ATG suppression, which relates to key biological properties, such as increased self-renewal, marked proliferation, and invasion. Hence, depressed ATG is a culprit in contributing to GSCs’ self-renewal and invasion within neighboring tissues. As a proof of concept, low levels of ATG are detected at baseline levels in GMB from patients in vivo, ex vivo, and in patients’ cell cultures [22,70,71,72,73]. The same occurs in experimental models including GBM xenografts and GBM cell lines [69,72,74,75,76,77]. In experimental settings, rescuing ATG associates with an inhibition of cell proliferation and invasion [52,69,72,74,77,78,79]. 

In this regard, various studies have shown that curcumin exerts anti-cancer activity by inhibiting mTOR signaling, which among various effects activates the ATG pathway [53,54,64,80,81,82]. Curcumin induces ATG trough the modulation of several upstream regulators of the mTOR pathway, such as the adenosine monophosphate-activated protein kinase (AMPK), the phosphatase and tensin homolog (PTEN)/Akt, the IκB kinase β (IKKβ), and the neural precursor cell expressed developmentally down-regulated protein 4 (NEDD4) [83,84,85]. Independently from mTOR upstream molecules, disruption of the mTOR–raptor interaction is another proposed mechanism of curcumin-mediated ATG activation [86]. In the next section, we provide an overview of the experimental studies centered on curcumin-mediated ATG induction as a strategy to combat GSCs in GBM.

## 3. Curcumin Suppresses GSCs’ Tumorigenicity through ATG Induction

Compelling evidence indicates that curcumin and curcuminoids counteract GBM malignant phenotype and aggressiveness by targeting ATG within GSCs. In this way, curcumin modulates GSC cell biology, including proliferation, viability, migration, and invasion [55,87,88].

Several in vitro studies reported that curcumin effectively prevents GBM cell proliferation through the perturbation of the mTOR pathway. For instance, Aoki et al. (2007) [81] demonstrate that curcumin induces G2/M cell cycle arrest through the induction of mTOR-dependent ATG in two human malignant glioma cell lines, namely U87-MG and U373-MG. This was evidenced by an increase in LC3 immunoblotting, enhanced red fluorescence using flow cytometry after acridine orange (AO) staining, and the ultrastructure of ATG vacuoles. Conversely, administration of the ATG inhibitor 3-methyladenine (3-MA) or a recombinant full-length human active Akt1 protein (rAkt1) suppresses the anti-tumor effects of curcumin [81]. Similar results were documented by Maiti et al. (2019) [56] in both mouse (GL261) and rat (F98). In these species, the levels of ATG markers within glial tumor cells (i.e., Atg5, Atg7, Beclin-1, LC3A/B, and p62) and the number of ATG vacuoles were increased following curcumin administration [56]. It is noteworthy that levels of PI3Kp85, p-PI3Kp85, total Akt, p-Akt, mTOR, and p-mTOR were decreased following curcumin administration. This indicates that this natural polyphenol significantly activates the mTOR-dependent ATG pathway [56]. This is in line with findings of Zanotto-Filho and colleagues (2011) [49,89], who found that curcumin inhibits the constitutive activation of the PI3K/Akt/mTOR pathway. This significantly reduces in vitro GBM cell viability. Intriguingly, curcumin does not modify the phenotype of health astrocytes, suggesting that this natural compound selectively targets glioma cells [49,89]. A curcumin-mediated increase in ATG levels occurs along with the down-regulation of cell survival markers such as Bcl-2 and the cytoprotective mitochondrial protein bcl-x. This is likely to depend on the cytostatic/anti-proliferative effect of curcumin on GBM cells [49,56,89].

**Figure 2:**
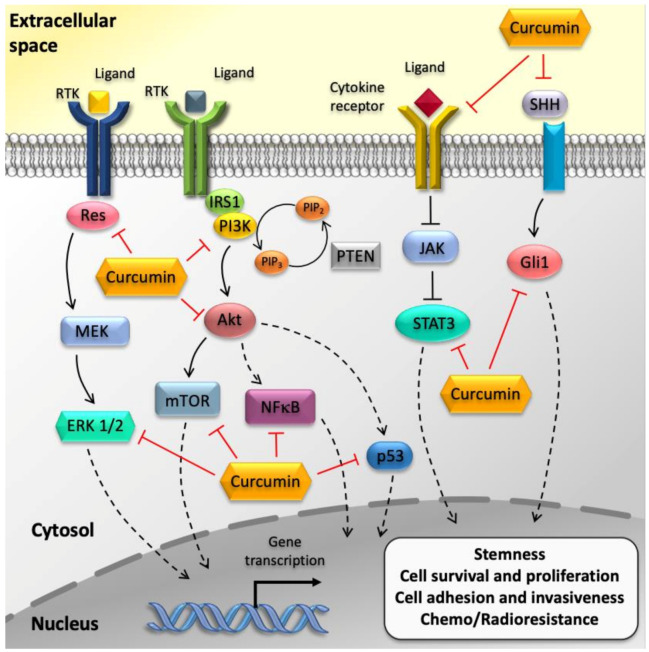
Effects of curcumin on GBM cancer stem cells (GSCs). The cartoon summarizes the major effects of curcumin on GSCs. In particular, curcumin was shown to decrease malignant characteristics of GSCs by targeting core-signaling pathways such as PI3K/Akt/mTOR, JAK/STAT3, and MAPK pathways.

Enhancing ATG in GSCs following curcumin administration produces multiple effects, well beyond a mere arrest of the cell cycle. For instance, curcumin suppresses the tumorigenic stem-like features and the invasiveness of GSCs by down-regulating Akt/mTOR activity (Figure 2). This is remarkable, since ATG defect contributes to the desensitization of GSCs to normal differentiation cues, and GSC also increased the invasive potential and therapeutic resistance [69,82,90].

In line with this, Zhuang et al. (2012) [52] provided evidence that curcumin suppresses GSC stem-like features, while triggering the ATG-dependent differentiation of GSCs both in vitro and in vivo [52]. In detail, the up-regulation of neural markers (i.e., βIII-tubulin, Tuj1, Olig2) and the marked reduction of GSC self-renewal and clonogenic ability occurs concomitantly with ATG induction following curcumin administration. Such an ATG-dependent pro-differentiating effect was replicated in vivo in nude mice bearing intracranial GBM xenografts. In fact, tumor sections obtained from curcumin-treated mice feature a remarkable increase in the number of LC3 immunofluorescent puncta and autophagosomes at transmission electron microscope. Notably, all these effects were accompanied by a marked reduction of tumor burden and increased mice survival. Consistently with the in vitro data, the dual effect of curcumin on GSC stemness and differentiation in xenograft tumors were reversed following treatment with 3-MA [52].

One of the major factors contributing to GBM malignancy is the highly invasive potential of GSCs. Unlike non-stem tumor cells, GSCs easily migrate and infiltrate within the surrounding healthy brain parenchyma [57]. It is known that hyper-activation of the Akt/mTOR pathway sustains the extremely invasive phenotype of GSCs, while mTOR inhibition down-regulates both mRNA, protein levels, and the activity of the matrix metalloproteinases (MMPs) MMP-9 and MMP-2, which promote tumor invasion through extracellular matrix degradation [69]. As a proof of concept, mTOR-dependent ATG induction following curcumin administration significantly impairs GSCs migration in vitro, as well as their ability to invade the brain parenchyma in vivo [49,58,64]. For instance, Zhang et al. (2016) [64] reported that curcumin-loaded nanoparticles (Cur/LDH NPs) significantly reduce cell migration and invasion in a A172 glioma cell line. These effects were associated with mTOR-dependent ATG stimulation. In fact, the number of ATG vacuoles and expression levels of ATG markers (LC3A/B, Atg5-Atg12) were increased in A172 cells exposed to Cur/LDH NPs [64]. Again, curcumin-induced down-regulation of the oncogenic protein NEDD4 impairs the migration of highly invasive SNB19 and A1207 GBM cell lines [91]. Notably, the NEDD4 is an E3-ubiquitin ligase, which promotes ubiquitin-mediated PTEN degradation, and thus PI3K/Akt activation and cell proliferation [91,92], and it is frequently overexpressed in various cancers, including GBM [92]. Collectively, these data confirm that the effects of curcumin on GSCs are tightly bound to ATG induction, and they further strengthen the notion that the ATG-dependent differentiation, migration arrest, and occluded invasion induce by curcumin may be promising in the adjunct therapeutic of GBM.

In line with this, curcumin derivatives exert beneficial effects on GBM due to their potential to suppress the tumorigenic features of GSCs [63,65,93,94]. Increasing evidence demonstrates that these synthetic compounds decrease cancer stem cell-like phenotype as well as invasive potential by targeting upstream regulators of the mTOR signaling in GSCs. For instance, hydrazinobenzoylcurcumin (HBC) suppresses self-renewal ability, cell migration, and invasion by down-regulating the Ca^2+^/CaM-dependent protein kinase II (CaMKII)/c-Met axis, which, in turn, modulates the expression of stemness markers through the activation of Akt/mTOR signaling [65].

Furthermore, curcumin sensitizes GSCs to several chemo- and radio-therapeutic agents due to its ability to modulate different cell signaling, beyond ATG [18,45,89,95,96,97,98]. In fact, when administered in combination with currently used therapies, curcumin further potentiates their anti-tumor activity against GBM [19,99,100,101]. For instance, curcumin enhances temozolomide (TMZ)-induced cytotoxicity by disputing the Akt/mTOR pathway in U87MG cell lines, thereby overcoming GBM therapeutic resistance [18]. Conversely, co-treatment with ATG inhibitors (i.e., 3-MA, hydroxychloroquine (HCQ), or LY294002) attenuates the antitumor effects of curcumin on human GBM cells, leading to an increased resistance to antitumor agents [80]. 

Finally, it is worth mentioning that mTOR alterations and defective ATG are also bound to several non-tumoral cells, which support tumor growth and mediate GBM relapse and infiltration [69]. These represent the micro-environment that borders tumor growth and promotes GSCs proliferation. This is the case of endothelial cells (ECs), which reinforce GSCs stem-like phenotype through the mTOR pathway [69,102]. Remarkably, curcumin markedly decreases in vitro tube formation and cell migration of rat brain capillary endothelial cells (RBE4) as well as in vivo blood vessel formation in mice bearing GBM xenografts, thus supporting an anti-angiogenic activity of curcumin in GBM [60]. Likewise, curcumin targets GBM-associated microglia, which is key in GSC immune evasion. In fact, these immune cells are recruited by GSCs through mTOR-dependent extracellular vesicle (EVs) release [103]. This process is quite powerful in the natural course of GBM and it consists of the release of GSC-derived EVs, which harbor diffusible pro-oncogenic cargoes. Once released within the extracellular milieu, these tumor-promoting EVs disseminate as paracrine factors to induce phenotypic and epigenetic modifications in glioma-associated stromal cells. In this way, GSCs actively recruit different non-tumorigenic stromal cells, which, in turn, participate in tumor micro-environment (TME) adaptive remodeling and immune modulation, which foster tumor growth and progression [103]. At the molecular level, recent publications revealed a potential role of mTOR-dependent ATG in modulating EV-mediated intercellular communication within GBM TME. In fact, there is emerging evidence that ATG and EV release are tightly intermingled and reciprocally regulated [104,105,106]. Hence, it is not surprising that mTOR hyper-activation and subsequent ATG suppression may profoundly alter the EV-dependent release of cytosolic cargoes, which intensely occurs in GBM. This is in line with the observation that GSCs secrete significant amounts of EVs enriched in proteins, mRNAs, and miRNAs, which, in turn, enable an unconventional mechanism of EV-mediated disease spreading [107,108]. Within this frame, EV-dependent cell-to-cell communication has been implicated in GSC-driven immune escape. Intriguing reports demonstrate that GSC-derived EVs act on monocytes, inducing a shift toward an immune-suppressive, tumor-supporting M2 macrophage phenotype, which in turn fosters tumor invasion and progression [103,109,110]. Remarkably, a recent in vivo study revealed that curcumin induces the repolarization of tumor-supporting M2-like microglia/macrophages toward the tumoricidal M1-like phenotype and intra-GBM recruitment of activated natural killer (NK) cells [111]. Notably, these effects were associated with a suppression of GSC and decreased tumor volume [111]. Although the specific molecular mechanisms have yet to be explored, novel insights on how curcumin-mediated ATG induction can reshape the GBM tumor micro-environment represents a novel field worth being deeply investigated. 

## 4. Effects of Curcumin on GBM beyond ATG Induction

In the last decades, curcumin received considerable interest in anti-cancer research, and especially in GBM, due to its versatile bioactivity via the modulation of several pathways, beyond ATG. In fact, at the molecular level, curcumin exerts pleiotropic effects by influencing a number of cellular signaling pathways, which are involved in tumor initiation, growth, and progression [112]. For instance, curcumin suppresses tumor growth by inhibiting tumor-promoting pathways, such as the NF-kB, Wnt/β-catenin, Notch signaling, as well as the JAK/STAT3 and MAPK pathways [45,101,113,114,115]. At the same time, curcumin up-regulates major tumor-suppressing proteins, namely p53, p21, and caspase 3 [46,116,117]. 

### 4.1. Curcumin Suppresses GBM Cell Proliferation and Survival via Inhibition of NF-κB and AP-1 Pathways

NF-κB is a transcription factor, which regulates the gene expression of several target genes involved in major cellular and biological processes, such as proliferation, survival immune response, and inflammation [118]. NF-κB is constitutively over-expressed in GBM [119], and its aberrant activation is linked to de-regulated, tumor-promoting EGFR and PI3K/Akt/mTOR signaling [120,121]. Similarly, the constitutive over-activation of c-Jun N-terminal kinase (JNK)/AP-1 transcription factor is associated with GBM growth, infiltration, and therapeutic resistance [45]. In fact, AP-1 is an upstream modulator of MMPs gene expression, thereby regulating GBM invasive potential [122].

In vitro studies, using both human (T98G, U87MG, T67, U373) and rat (C6) GBM cell lines, demonstrated that curcumin and curcuminoids (i.e., demethoxycurcumin (DMC)) reduce cell survival through the suppression of both NF-κB and AP-1 signaling activation. This prevents the constitutive activation of Akt and JNK [45,100,101,123,124]. At the same time, the curcumin-dependent inhibition of AP-1 signaling suppresses MMPs transcription, thus markedly repressing GBM cells invasive potential [122]. These results are in line with research reports demonstrating that the curcumin-dependent suppression of MMP-9 promoter activity occurs via the inhibition of NF-κB and AP-1 DNA binding activities [125]. Furthermore, the inhibition of NF-κB signaling with curcumin potentiates the anti-tumor effects against GBM of different therapeutic agents such as the alkylating agent nimustine hydrochloride (ACNU) [101] and the microtubule-stabilizing agent paclitaxel (PTX) [100].

### 4.2. Curcumin Induces GBM Cell Cycle Arrest through the Modulation TP53 and RB Pathways

Compelling evidence demonstrates that curcumin exerts anti-proliferative effects on glioma cells by modulating TP53/MDM2/MDM4/p14ARF and RB1/CDK4/p16INK4A signaling. These latter represent two main cell cycle regulating pathways, which are often impaired in GBM [44]. Remarkably, curcumin significantly inhibits GBM cell growth and proliferation via the suppression of cell cycle progression in different human glioma cell lines [19,47,81,113,126,127]. For instance, Liu et al. (2007) [47] demonstrate that curcumin induces G2/M cell cycle arrest in a p53-dependent manner. In fact, p53 protein levels are increased in curcumin-treated U251 glioma cells, followed by the induction of CDK inhibitor/cell-cycle regulator p21 and tumor suppressor ING4, thus resulting in cell cycle arrest [47]. Similarly, treatment with curcumin up-regulates p53 and p21 expression while suppressing the cdc2 and RB pathways in DBRTG glioma cells [113]. Moreover, another study in U87MG cells shows that curcumin induces GBM cell-cycle arrest through the up-regulation of p21, along with cyclin D1 down-regulation. Remarkably, this occurs independently from p53 function, since it relies on the activation of Egr-1 transcription factor via ERK and JNK/MAPK/Elk signaling cascades. In fact, curcumin-induced p21 transcription is abolished in human U87MG cells transfected with Egr-1 siRNA [117]. In the attempt to unravel the molecular mechanisms underlying curcumin-mediated growth inhibition, a recent paper reported that U251-treated cells are arrested in the G2/M phase by increased expression of the tumor suppressor death-associated protein kinase 1 (DAPK1) [127]. Interestingly, this effect is accompanied by the inhibition of NF-κB and STAT3 pathways, along with caspase 3 activation. In fact, siRNA-mediated knock-down of DAPK1 attenuates curcumin-induced inhibition of NF-κB and STAT3 while preventing caspase 3-mediated apoptosis [127].

### 4.3. Curcumin Hampers GBM Aggressiveness through the Modulation of The JAK/STAT Pathway

A recent report demonstrates that curcumin hampers GBM aggressiveness in vitro by inhibiting the JAK/STAT3 pathway [128]. In fact, curcumin potently suppresses GBM cell proliferation through the modulation of the JAK/STAT3 pathway both in GBM human primary and recurrent cell lines [48]. In detail, the anti-proliferative effect was associated, at least in part, with a reduction of STAT3 intracellular levels, resulting in the reduced transcription of cell cycle regulating gene c-Myc and proliferation marker Ki-67 [48].

In addition to anti-proliferative effects, curcumin-mediated inhibition of JAK/STAT3 signaling strongly correlates with a marked suppression of GBM cell migration and invasive potential [48]. Similarly, DMC and TMZ synergistically inactivate JAK/STAT3 signaling in human GBM cells lines, which accounts for a significant inhibition of cell proliferation and concomitant increase in apoptosis [62].

### 4.4. Curcumin Induces Pro-Apoptotic Pathways in GBM Cells

Among the pleiotropic activities exerted by curcumin, the anti-proliferative effects of this natural polyphenol rely, at least in part, on the activation of pro-apoptotic pathways. In fact, several studies demonstrate that curcumin-induced G2/M cell cycle arrest frequently occurs along with an induction of caspase-mediated cell death [46,123,127,129]. Remarkably, curcumin induces apoptosis via a caspase-dependent pathway in human GBM cells [46,130]. In fact, treatment with curcumin increases the expression pro-apoptotic proteins, namely caspase-3, caspase-7, caspase-8, and caspase-9, which initiate and execute the apoptotic cascade [46,130]. Although the molecular mechanisms are yet to be explored, curcumin triggers several pro-apoptotic effects, beyond an increase in caspase activity. In fact, curcumin induces DNA fragmentation as well as a cleavage of poly(ADP-ribose) polymerase (PARP-1) nuclear enzyme in human glioma CHME cells [131]. Furthermore, a loss of mitochondrial membrane potential and ROS production are increased in curcumin-treated cells compared with controls [131]. This suggests indeed the induction of the apoptotic cascade. At the same time, curcumin exerts anti-GBM activity by suppressing anti-apoptotic signals [45,126], as confirmed by an increased BAX:BCL2 ratio in several human GBM cell lines [50,130,131]. It is noteworthy that these effects are related, at least in part, to the inhibition of sonic hedgehog (SHH)/glioma-associated oncogene homolog 1 (GLI1) signaling, resulting in down-regulation of the downstream oncoprotein Bcl-2 [59].

## 5. Concluding Remarks

Extensive research in the past two decades demonstrates the beneficial effects of phytochemicals in general, and especially curcumin, in a wide range of human diseases, encompassing NDDs and brain tumors, and particularly GBM. In fact, curcumin supplementation provides neuroprotection due to its anti-oxidant, anti-inflammatory and, anti-protein aggregation properties, which are tightly bound to ATG modulation. Furthermore, mounting evidence demonstrates that curcumin is capable of targeting undifferentiated and highly tumorigenic cancer stem cells, and especially GCSs, through the modulation of the mTOR-dependent ATG pathway. Notably, mTOR hyper-activation and defective ATG are both implicated in the maintenance of GSCs’ oncogenic properties and metabolism. Conversely, rescuing ATG with curcumin suppresses the tumorigenic features of GSCs, thus counteracting GBM establishment and growth. Since curcumin influences various aberrant signaling pathways associated with GBM, and especially mTOR-dependent ATG, it should be further exploited as a potential adjunct therapy for GBM standard treatments.

### Outstanding Questions

Even though the hydrophobic nature, low water solubility, and physicochemical stability may limit its bioavailability [132], increasing evidence demonstrates that curcumin is able to cross the blood-brain barrier, thus yielding therapeutic benefits within the CNS [133]. However, extensive efforts are currently devoted to develop novel delivery systems (i.e., nanoparticles, liposomes, micellar systems, combination with piperine) [134,135,136] as well as curcumin derivatives [137,138], thus possibly overcoming these obstacles, while improving curcumin’s efficacy.

## Figures and Tables

**Table 1:** In vitro anti-tumor effects of curcumin on glioblastoma multiforme.

Cell Line (s)	Dose (s)	Molecular Target (s)	Effect (s)	Reference
T98G *, U87MG *, T67 *, C6 ^‡^	25–50 μmol/L	AP-1, NFκB	Reduces cell survival; suppresses chemotherapy resistance	[44]
U251 *	10 μM	p53, ING4, p21 WAF-1/CIP-1	Inhibits cell growth, induces G2/M cell cycle arrest	[46]
A172 *, MZ-18 *, MZ-54 ^§^, MZ-256 ^§^, MZ-304 ^§^	10 μM, 20 μM and 50 μM	JAK/STAT3	Inhibits cell proliferation, migration, and invasion	[47]
U251 *, SNB19 *	10 μM, 15 μM	pAkt, p57, Skp2	Inhibits cell proliferation, migration, and invasion, induces cell cycle arrest and apoptosis	[48]
U87MG*	25 μM, 50 μM	NFκB, IAPs, Smac/Diablo, Bax, Bcl-2, caspase-3	Decreases cell viability and induces apoptosis	[49]
U87 *, U251 *	2.5 μM (IC_50_ 25 μM)	STAT3, MAPK IAP, ROS,	Decreases cell viability, inhibits proliferation, sphere-forming ability, and colony-forming potential of glioblastoma stem cells	[50]
SU-2 *, SU-3 *	2 μM	GFAP, Tuj1, Olig2, βIIItubulin, LC3	Induces ATG and differentiation, while inhibiting self-renewal in glioma-initiating cells (GICs)	[51]
U87 *	10 µM/L, 20 µM/L	STAT3	Inhibits cell migration and invasion	[52]
A172 *	10 μM	Atg5, Beclin-1	Induces ATG	[53]
U87-MG *, U373-MG *	20 μM, 40 μM	Akt/mTOR/p70S6K, ERK1/2	Induces G2/M arrest, inhibits cell growth, induces ATG	[54]
U-87MG *, GL261 ^†^, F98 ^‡^, C6 ^‡^	25 μM	Atg5, Atg7, Beclin-1, LC3A/B, p62, PI3K/Akt/mTOR	Induces ATG	[55]
U87 *, U373 *, U138MG *, C6 ^‡^	7.5 μM, 10 μM and 15 μM (IC_50_ 19–28 μM)	PI3K/Akt, NFκB, caspase-3	Induces G2/M cell cycle arrest, inhibits cell proliferation	[56]
U251 *, U87 *	10 μM, 20 μM and 40 μM	p-Akt, p-mTOR, PTEN, p53	Inhibits cell proliferation, migration, and invasion, while inducing apoptosis	[57]
SNB19 *, A1207 *	10 μM, 15 μM and 20 μM	PI3K/Akt, Notch1, NEDD4	Inhibits cell proliferation, induces cell cycle arrest, inhibits cell migration and invasion	[58]

* Human glioma cell lines; ^§^ Human recurrent GBM cell lines; ^†^ Mouse glioma cell lines; ^‡^ Rat glioma cell lines.

**Table 2:** In vivo anti-tumor effects of curcumin on glioblastoma multiforme.

Model (s)	Cell line (s) and Injection Site	Dose (s)	Effect (s)	Reference
Intracranial xenograft	U-87; caudate-putamen	i.p. injection (120 mg/kg)	Increases survival of curcumin-treated mice	[59]
Intracranial xenograft	SU-2 and SU-3; caudate nucleus	i.p. injection (300 mg/kg)	Increases survival of curcumin-treated mice	[51]
Subcutaneous injection	U87MG; right flank	Intratumoral injection (100 mg/kg)	Inhibits tumor growth and induces ATG	[54]
Intracranial xenograft	C6; striatum	i.p. injection (50 mg/kg)	Inhibits tumor growth	[56]
Subcutaneous injection	U87; flank	i.p. injection (60 mg/kg)	Decreases tumor volume	[57]
